# Supplementary material for: A Model for the Diversity Explosion Fundamental to Metastasis
Source: bioRxiv. 2025 Dec 16:2025.12.13.694153. Preprint. [Version 1] doi: 10.64898/2025.12.13.694153 (PMC12724465; doi:10.64898/2025.12.13.694153)
Supplement: Supplement 1 [file NIHPP2025.12.13.694153v1-supplement-1.pdf]

bioRxiv preprint doi: https://doi.org/10.64898/2025.12.13.694153; this version posted December 16, 2025. The copyright holder for this preprint (which was not certified by peer review) is the author/funder, who has granted bioRxiv a license to display the preprint in perpetuity. It is made available under aCC-BY 4.0 International license.

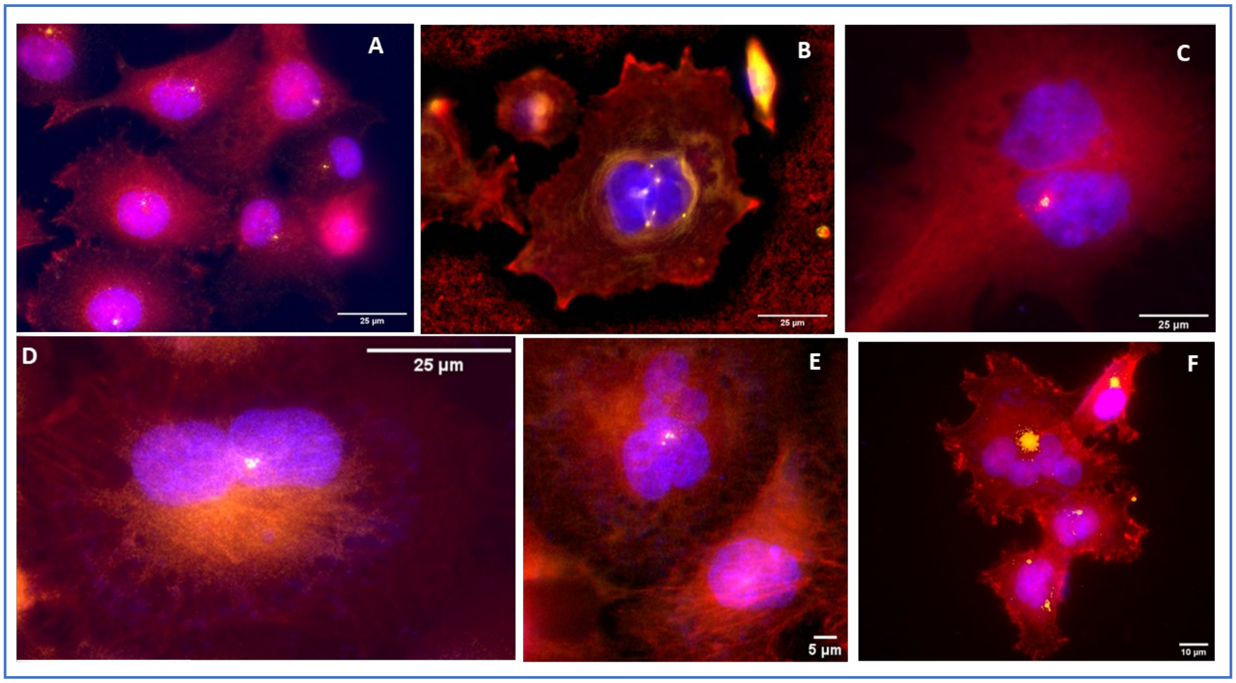

Supplemental Figure 1. Fixed control and ALK 4 KO multinuclear cells stained for F-actin (red), DNA (nuclei; blue) and for centrosomes (yellow). Antibodies to pericentrin were used to locate the centrosomes in these widefield microscopy images. Control (A) EV cells in interphase displayed the one centrosome/cell expected for this phase of the cell cycle. In contrast, the ALK 4 KO multinuclear cells showed a much more random number and location of these critical organelles (B-F). Note that these images were manipulated in order to enable the visualization of the centrosomes. Nothing was pasted in or moved relative to anything else in the image. The main tool was shade correction and contrast adjustment.

bioRxiv preprint doi: https://doi.org/10.64898/2025.12.13.694153; this version posted December 16, 2025. The copyright holder for this preprint (which was not certified by peer review) is the author/funder, who has granted bioRxiv a license to display the preprint in perpetuity. It is made available under aCC-BY 4.0 International license.

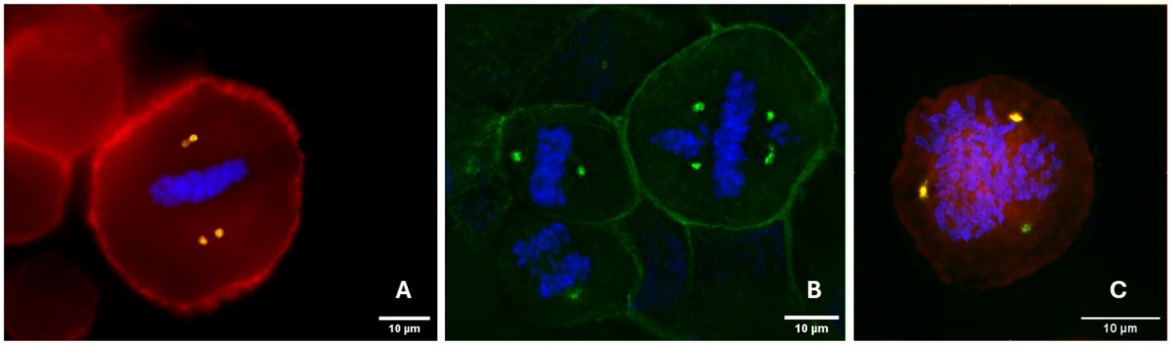

Supplemental Figure 2. Fixed control and ALK 4 KO multinuclear cells stained for F-actin (red), DNA (nuclei; blue) and for centrosomes (yellow). Structured illumination microscopy was used to image cells that had been blocked in mitosis. Examples of three configurations are shown; each would again shuffle the chromosomes in unique ways, but many other patterns are probable.

bioRxiv preprint doi: https://doi.org/10.64898/2025.12.13.694153; this version posted December 16, 2025. The copyright holder for this preprint (which was not certified by peer review) is the author/funder, who has granted bioRxiv a license to display the preprint in perpetuity. It is made available under aCC-BY 4.0 International license.

Supplemental Movie Legends

Movies published to figshare, DOI 10.6084/m9.figshare.30782168

Legend to Supplemental Movie 1. Colony formation by ALK 4 KO Panc 1 single cell clones showing diversity in morphology and behavior. Cells were plated through 8 µm pore uncoated transwell filters (Corning, inc) directly onto 12 well multiwell tissue culture plastic that had been treated with 0.01 mg/mL Matrigel (no growth factors). Normal 10% serum medium was present on both sides of the filter. After 24-48 hrs the filter wsa removed, medium replaced, and the cells allowed to grow for 14 days. Time lapse recordings were obtained using a Zeiss Live Cell Station with 5% CO2 and 37 deg at intervals of 4 min. Images were at 5X brightfield to a CoolSnap 1392x1040 pixel CCD camera (Photometrics; Roper Scientific).

Legend to Supplemental Movie 2. ALK 4 KO cells that were able to pass through 3 µm transwell-type filters (Genesse Scientific) directly onto Matrigel- coated tissue culture plastic, were imaged at 4 min intervals, as described for Supplemental Movie 1. They showed motility and poor adhesion once cell division had take place.

Legend to Supplemental Movie 3. Large motile multinuclear cell shows rapid motility, and the property of transporting much smaller cells for long distances. Cells were grown from cells that had passed through the 3 µm filters, and imaging as described in Supplemental Movie 1.

Legend to Supplemental Movie 4. Two cells (yellow arrow) appear to merge together and proceed as one cell. ALK 4 Ko cells that had passed through 3 µm filters were imaged as described in Supplemental Movie 1.

Legend to Supplemental Movie 5. ALK 4 Ko cell rounds up to begin mitosis, pauses for several hours, and then does not finish cytokinesis and moves away as a binuclear cell. Imaging was carried out described as for Supplemental Movie 1. Slices from this movie are presented in Figure 10.

Legend to Supplemental Movie 6. ALK 4 Ko cell in center of frame round up and divides into three daughters. The daughters then move away independently. Imaging was carried out described as for Supplemental Movie 1.

Legend to Supplemental Movie 7. Movie basis for Figure 11; Large cell divides into three daughters. Interesting also for the “triskelion” configuration of chromosomes achieved at one point in the process. Imaging was carried out described as for Supplemental Movie 1.

bioRxiv preprint doi: https://doi.org/10.64898/2025.12.13.694153; this version posted December 16, 2025. The copyright holder for this preprint (which was not certified by peer review) is the author/funder, who has granted bioRxiv a license to display the preprint in perpetuity. It is made available under aCC-BY 4.0 International license.

Legend to Supplemental Movie 8. Movie basis for Figure 12; Large ALK 4 KO multinuclear cell with many small nuclei, breaks up into many smaller cells in a very aberrant version of cytokinesis. Many of the daughters then begin to undergo cell division. Imaging was carried out described as for Supplemental Movie 1.

Legend to Supplemental Movie 9. A second example of an large multinuclear cells breaking up into many smaller cells without undergoing a standard cytokinesis process. This movie was obtained at 10X phase contrast initial magnification, but otherwise the same time lapse conditions as Supplemental Movie 1.
